# Case Report: Mixed-Cause Vertigo and Sudden Sensorineural Hearing Loss as Presentations of Vertebrobasilar Dolichoectasia

**DOI:** 10.7759/cureus.28136

**Published:** 2022-08-18

**Authors:** Carolina Berenice Anízar Rodríguez, Dulce Maria Mendoza Ugalde, Rodolfo A García-Tecpa

**Affiliations:** 1 Department of Audiology, Otoneurology, Hospital de Especialidades del Centro Médico Nacional Siglo XXI, Mexico City, MEX

**Keywords:** aica, hints, vertigo, dolichoectasia, sudden sensorineural hearing loss

## Abstract

Sudden sensorineural hearing loss (SSNHL) is a syndrome characterized by rapid progression of hearing impairment over seconds to days. While no universally accepted definition exists, it is often defined as a sensorineural hearing loss of 30 decilbles (dB) or more across at least three contiguous frequencies, occurring within 72 hours.

In elderly patients and those with vascular risk factors who develop SSNHL, ischemia of the vertebrobasilar territory is suspected, especially of the anteroinferior cerebellar artery. This is because ischemia that affects this artery produces 79% of SSNHL associated with cerebral infarction. In many cases of ischemia of the vertebrobasilar territory, there is an association with anomalies of the vertebrobasilar circulation, such as vertebral artery hypoplasia or vertebrobasilar dolichoectasia.

Here, we report a case of a 73-year-old man who presented right sudden hearing loss accompanied by acute onset vertigo, a physical exploration compatible with right vestibular dysfunction, and a history of recurrent episodes of syncope in the context of vertebrobasilar dolichoectasia, as diagnosed via magnetic resonance imaging (MRI).

## Introduction

Sudden sensorineural hearing loss (SSNHL) is a syndrome characterized by a rapid progression of hearing loss in the span of seconds to days. There is no universally accepted definition, but it is frequently defined as a sensorineural hearing loss of 30 decibels (dB) or more in three or more consecutive frequencies, occurring in less than 72 hours [1].

The incidence of SSNHL, secondary to ischemia, in the territory of the vertebrobasilar circulation is very rare, and in a series of 364 cases, it corresponded to 8% of them [2]. In elderly patients, and with risk factors for vascular disease, who present with SSNHL, ischemia in the territory of the vertebrobasilar circulation must be suspected, especially in the area of the antero-inferior cerebellar artery (AICA) which produces 79% of SSNHL related to cerebrovascular ischemic events [2].

The syndrome produced by occlusion of the AICA constitutes 0.9% of ischemic cerebrovascular events [2]. In many cases, it is associated with anatomic anomalies of the vertebrobasilar system, such as basilar artery hypoplasia and vertebrobasilar dolichoectasia. The most characteristic signs and symptoms include hypoacusia, tinnitus, vertigo, vomit, dysarthria, facial palsy, loss of sensitivity in the territory of the trigeminal nerve, Horner syndrome, homolateral appendicular dysmetria, and contralateral thermalgesia [2].

Vertebrobasilar dolichoectasia is incidentally diagnosed in a majority of cases; symptomatic patients can display both vascular and compressive signs and symptoms. Findings in affected patients can include transitory ischemic events, cerebrovascular ischemic events, subarachnoid hemorrhage, and compressive alterations of the brainstem and cranial nerves [3].

Here, we report a case of a 73-year-old man who presented right sudden hearing loss accompanied by acute onset vertigo, a physical exploration compatible with right vestibular dysfunction, and a history of recurrent episodes of syncope in the context of vertebrobasilar dolichoectasia, as diagnosed via magnetic resonance imaging (MRI).

## Case presentation

A 73-year-old man arrived at the hospital complaining about sudden onset right-side hearing loss accompanied by vertigo that had started three days before arrival, posterior to a syncopal event.

The patient has a previous history of hypertension under treatment with losartan and furosemide. Additionally, the patient complained about a three-year condition characterized by syncopal events which have produced multiple head injuries due to falling. The patient mentioned that the syncopal episodes occurred at a frequency of approximately twice or thrice weekly with the immediate recovery of consciousness, and that they are accompanied by simple visual hallucinations and sporadic episodes of vertigo that last under a minute. He denied other neurological symptoms.

**Figure 1:**
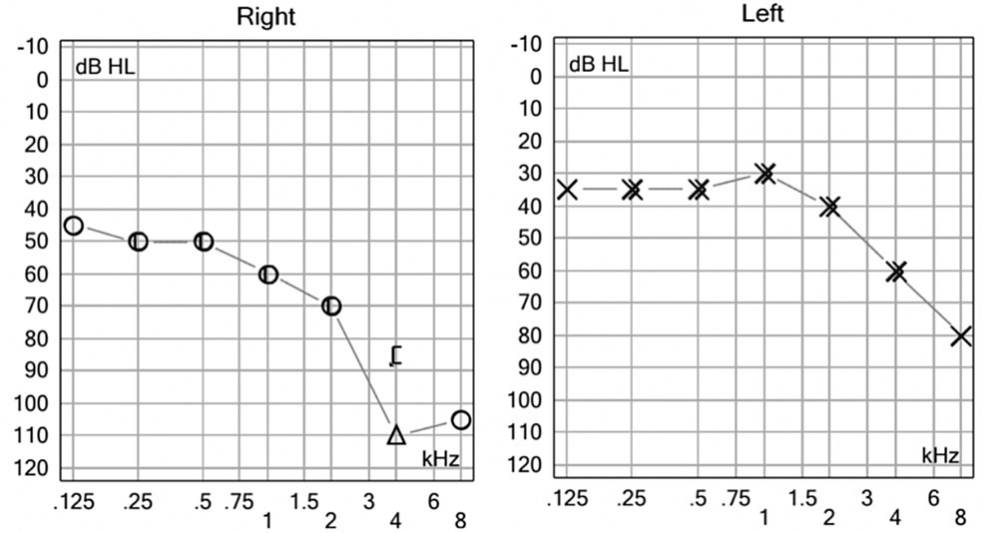
Initial Pure Tone Audiometry. We observed moderate (borderline severe) sensorineural hearing loss in the right ear. dB: decibel, HL: hearing level

**Figure 2:**
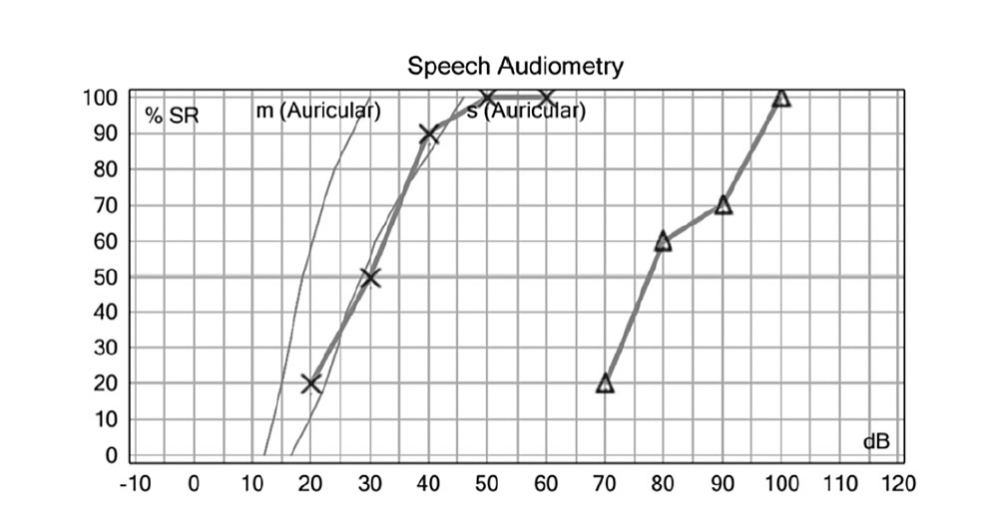
Initial Speech Audiometry. It shows maximum speech discrimination in the right ear of 100% at 100 decibels. dB: decibel

During physical exploration, we found a normal otoscopy and abnormal accumetry compatible with right side sensorineural hearing loss. Pure tone audiometry was performed finding results compatible with right SSNHL (Figures 1, 2).

An initial vestibular exploration utilizing HINTS (Head Impulse test, test of Nystagmus, Test of Skew) “plus” was performed with the following findings: 1) abnormal head impulse test on the right side; 2) horizontal spontaneous nystagmus to the left that does not change direction with eye movements; 3) normal test of skew.

Such findings are commonly accompanied by right side hypoacusis, as evidenced by the pure tone audiometry. The lateralization of the gait to the right and the exacerbation of the spontaneous nystagmus at the head shaking maneuver are coherent in the context of acute onset vertigo, produced due to vestibular dysfunction associated with homolateral SSNHL.

However, when continuing vestibular exploration, some findings were not coherent with these types of lesions. It was observed that the amplitude of the nystagmus did not change by directing the eye to the fast phase or to the slow phase, also no change in the amplitude of the nystagmus was observed with visual fixation. Additionally, the nystagmus changed direction during positional maneuvers (Dix-Hallpike, hyperextension, and roll test) in a direction that was different from the expected one of a patient with benign paroxysmal postural vertigo. 

**Figure 3:**
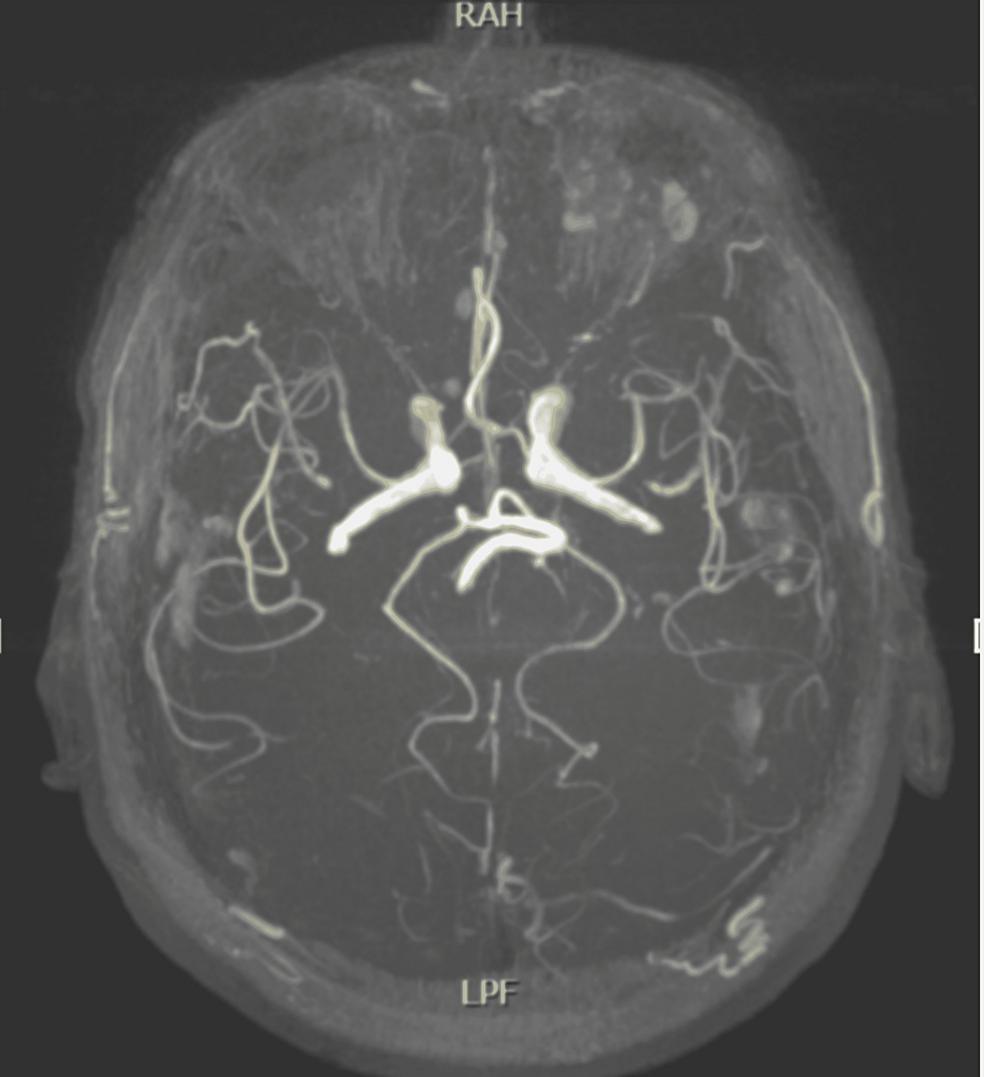
Brain MR-angiography. Right vertebrobasilar dolichoectasia.

In response to the finding of right SSNL, a dose-reduction oral steroid was initiated for 15 days. Concerning the history of repetitive syncopal episodes and the inconsistent findings in the vestibular exploration, an MRI was performed. The brain MR-angiography reported dolichoectasia of vertebrobasilar circulation at the level of the right vertebral artery in the V4 segment (Figure 3).

**Figure 4:**
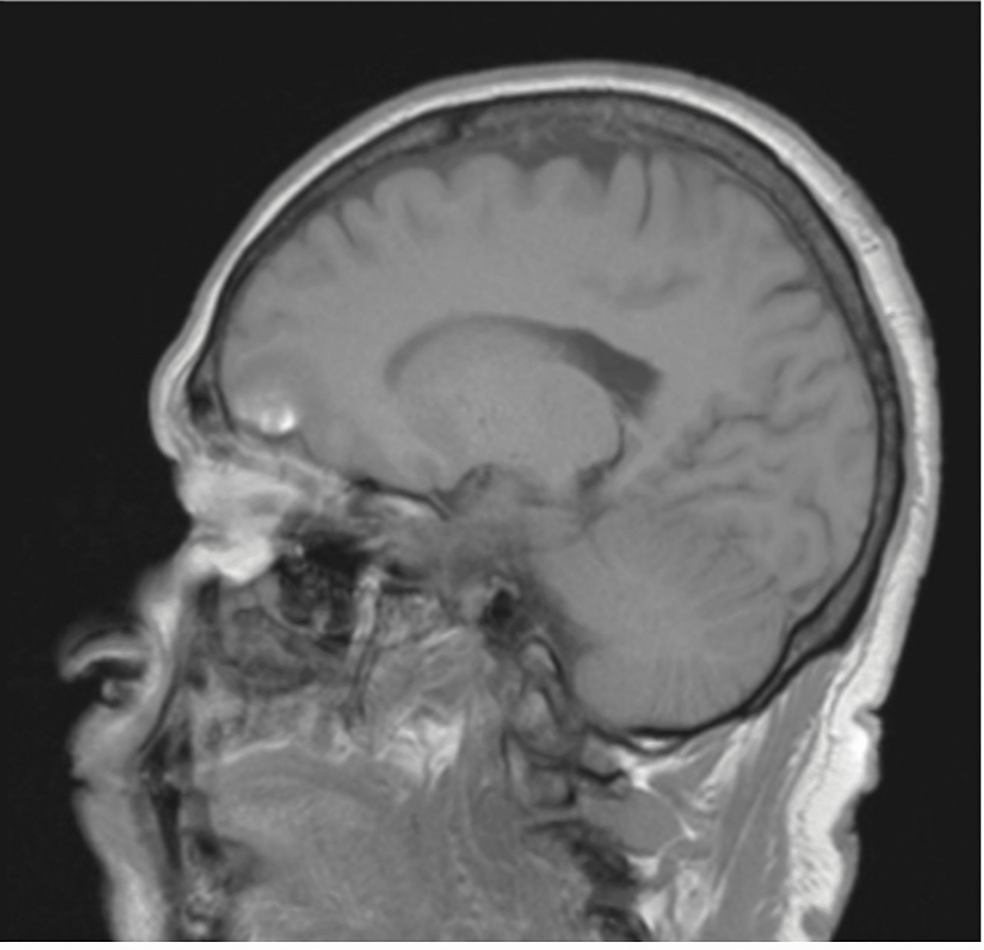
Sagittal T1 MRI Brain. Hyperintense lesion in frontal lobe suggestive of hemorrhage.

**Figure 5:**
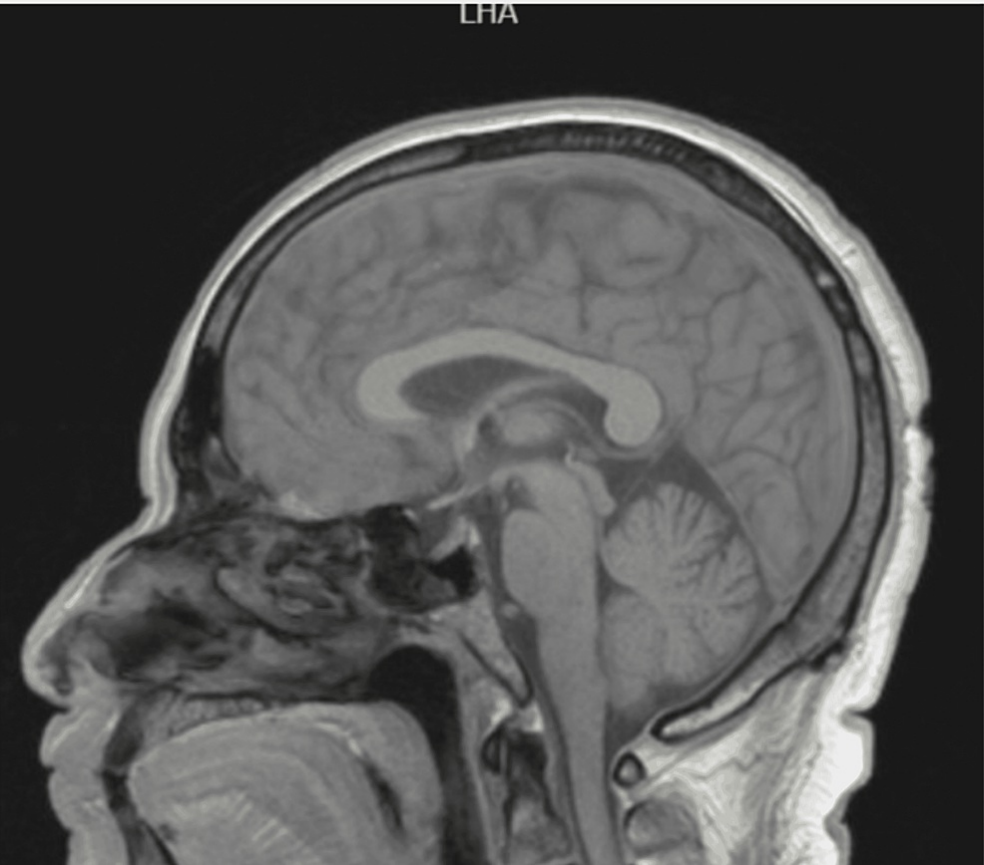
MRI Sagittal T1. MRI brain showing a cerebral arachnoidocele.

Besides this, the parenchyma was observed with generalized diminished volume and with hyperintense lesions in T1 and hypointense lesions in the fluid attenuated inversion recovery (FLAIR) of heterogeneous aspects, on both sides of the brain-the anterior is presumably related to frontal bilateral hematomas (Figure 4), secondary to brain trauma caused by repeat syncopal events. The same MRI demonstrated findings compatible with cerebral arachnoidocele (Figure 5).

**Figure 6:**
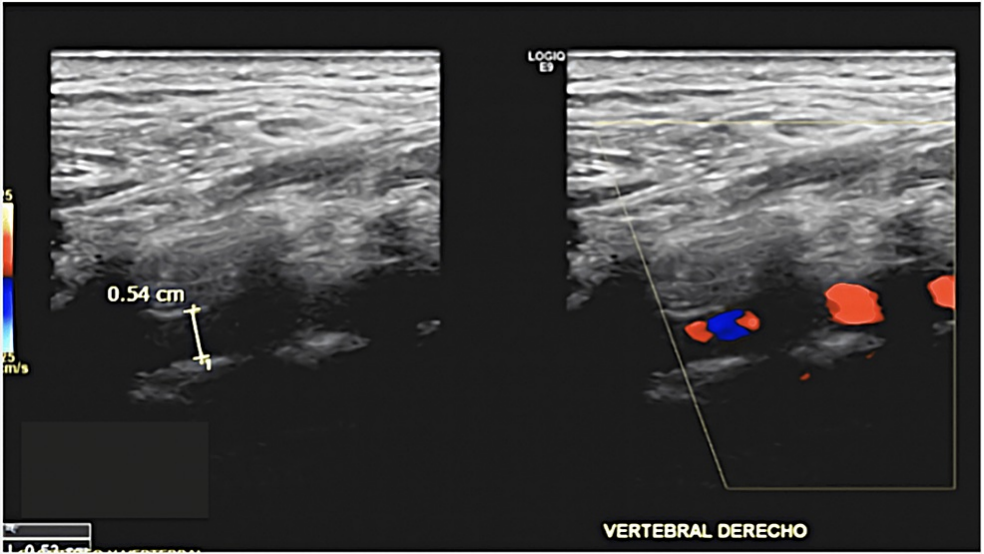
Vertebral Artery Doppler. Increased diameter of the right vertebral artery.

A carotid/vertebral doppler ultrasound was performed on the patient, and the test showed bilateral carotid artery dolichoectasia of the right vertebral artery (Figure 6).

**Figure 7:**
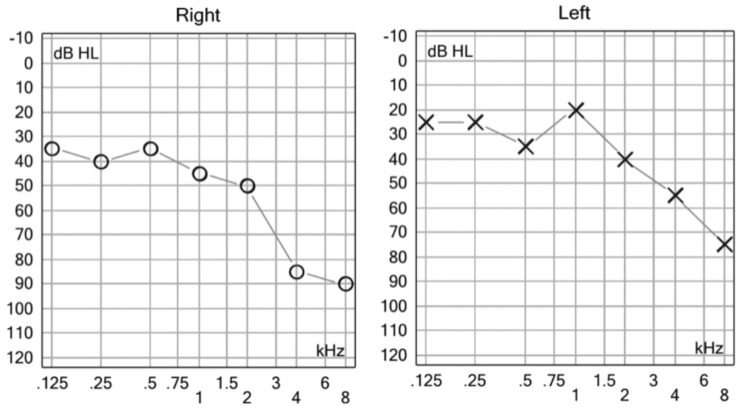
Tone Pure Audiometry after a Short Regimen of Oral Prednisone. dB: decibel, HL: hearing level

**Figure 8:**
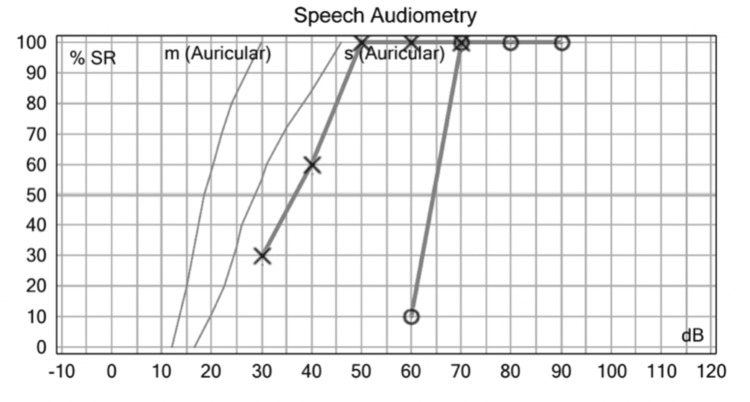
Final Speech Audiometry. Right maximum discrimination at 70 dB. We observed a final hearing level of 43 dB with a gain > 15 dB, which is considered a partial recovery according to Siegel's criteria. dB: decibel

The patient returned to our medical department after finishing the dose reduction treatment with oral prednisone, and a new pure tone audiometry was performed. The new test showed partial recovery (Figures 7, 8) according to Siegel’s criteria (Table 1). Following this, the patient continued multidisciplinary follow-ups with neurology, audiology, and otoneurology.

**Table 1:** Siegel’s Criteria. Siegel’s criteria of hearing improvement in sudden sensorineural hearing loss [4]. dB: decibel, PTA: pure tone average

Type	Hearing recovery
Complete recovery	Final PTA < 25 dB.
Partial recovery	More than 15 dB of gain. Final PTA between 25-45 dB.
Slight improvement	More than 15 dB of gain. Final PTA >45 dB.
No improvement	Gain <15 dB.

## Discussion

Dolichoectasia is defined as the abnormal dilation, elongation, and tortuosity of arterial vessels [3]. The reported prevalence is 0.05-0.06%, and the vertebrobasilar circulation is the most frequently affected; in contrast, the anterior cerebral circulation is rarely involved [5]. The precise etiology of dolichoectasia is still unknown, although it has been associated with many diseases such as systemic arterial hypertension, atherosclerosis, collagen-related diseases, polycystic kidney disease, and sickle cell anemia [3]. It is believed that the causative mechanism behind this disease is linked to an alteration of the balance of the activity between metalloproteinases and proteinases within the connective tissue of the arterial walls, thereby generating aberrant vascular remodeling and defective formation of the connective tissue of the arterial walls [3].

**Figure 9:**
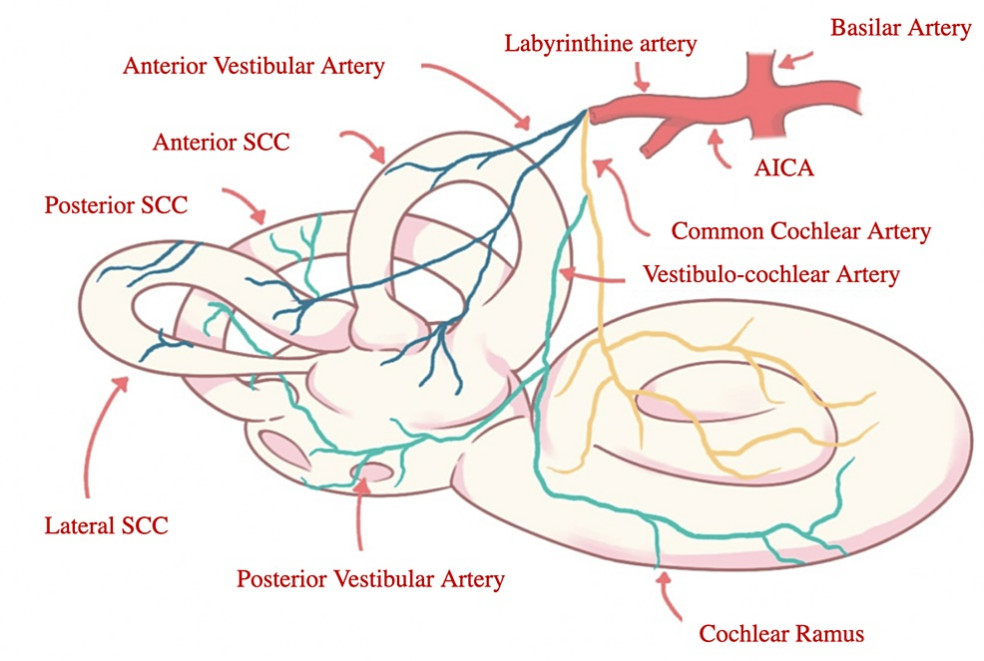
Inner Ear Arterial System. Image credits: Carolina Anízar. AICA: antero-inferior cerebellar artery, SCC: semicircular canal

The vertebrobasilar system is the primary arterial system in charge of the blood supply for the cerebellum and inner ear (Figure 9). Important arteries for cerebellar and inner ear irrigation emerge from this system. Such is the case of the AICA, which penetrates through the inner acoustic meatus and is in charge of the irrigation of the inner ear [6].

Since the arterial labyrinthic branches are small and have less collateral blood supply, it makes the inner ear especially vulnerable to the effects of ischemia in the posterior cerebral circulation [2].

It is unsurprising that any situation that threatens the normal functioning of the vertebrobasilar circulation can consequently produce cerebellar (instability, diplopia, ataxia, dysarthria, vertigo, and dizziness), audio-vestibular (vertigo, hypoacusia, tinnitus) and bulbar symptoms (transitory loss of consciousness due to affection of the ascending activating reticular system, which is in charge of keeping the person alert) [7].

Many neuroimaging studies, including diffusion MRI, may be unable to reveal small cerebrovascular events, especially in the posterior fossa [8]. It has been reported that 50% of false negatives can show up in the first 48 hours of the lesion occurring [9]. Something similar occurs with reference to the transitory ischemc attacks (TIA), making it impossible to make a direct observation of the affected region. This is also a possible reason why the MRI did not show alterations in this region in our patient. However, it is important to emphasize the correct diagnosis in these patients, because it has been corroborated that the risk of a stroke increases three-fold in the following four years in those who have had vertigo as the only presentation of posterior TIA [8].

HINTS plus is a diagnostic tool with a sensitivity of 99% and specificity of 97% that helps us make an accurate differentiation in vertiginous syndromes. It is widely used to identify and differentiate between sudden onset vertigo of peripheral etiology and central etiology, making it even more useful for the diagnosis of cerebrovascular ischemic events than diffusion MRI [9]. It is based on a four-step protocol: the head impulse test, identifying the characteristics of the nystagmus, the test of skew, and the presence of hypoacusis on the affected side [9]. It is termed “dangerous HINTS” when one or more of the following findings are identified: normal head impulse test, central characteristic nystagmus, and altered test of skew.

Hypoacusia can be caused by peripheral inner ear pathologies or infarct in the territory of the AICA [9]. Transitory ischemic attack of the AICA frequently causes mixed-origin vestibulopathy [8]. It is essential to mention that the HINTS protocol may not be solid enough to identify AICA infarction, because the head impulse test is positive in the majority of cases due to AICA’s role in the irrigation of the labyrinth [10].

In the case of our patient, initial HINTS plus was compatible with peripheral etiology. However, considering that the spontaneous nystagmus did not decrease with visual fixation - thereby not following Alexander’s laws - and because the nystagmus changed direction in the positional maneuvers in the absence of nystagmus compatible with the expected to be found in a patient with benign postural paroxysmal vertigo, an MRI was performed under suspicion of possible central etiology. On the other hand, in this patient, sellar arachnoidocele was also reported in the MRI.

Sellar arachnoidocele is an imaging finding in which it is possible to see a herniation of the subarachnoid space in to the hypofisiary cavity, thereby resulting in the entrance of the arachnoid and cerebrospinal fluid into this cavity [11,12]. According to its etiology, this can be classified as primary if there is no pathological process that causes hypofisiary damage. Insufficiency in the sellar diaphragm, increase in intracranial pressure, and reduction in the size of the hypophysis are causes of primary sellar arachnoidocele. Otherwise, secondary arachnoidocele is more frequent and is caused by pathological processes in the said region. Inside these mechanisms, we can include head trauma (such is the case in our patient), postpartum hemorrhage, thrombosis, meningitis, hypofisiary infarctions, surgical procedures, hydrocephalus, tumors, thrombosis, meningitis, and radiotherapy among other causes [11,12]. This finding can be asymptomatic or can exhibit an “empty sellar syndrome,” characterized by neurological, endocrine, ophthalmologic, audiologic, and vertiginous impairments [11,12]. Among the main symptoms associated with this pathology are cephalea, visual alteration (decreased visual acuity, alteration of the visual field, and hemianopia), depression, and hormonal alterations such as hyperprolactinemia and growth hormone deficiency [12].

Many studies show a correlation between sellar arachnoidocele and audiological and vestibular symptomatology. Out of these, the most important was made by Boleaga and Guzman, where the presence of vertigo was observed in 40% of the patients with sellar arachnoidocele [12]. Among the hypotheses for this, one proposes that an increase in the pressure enforced by the cerebrospinal fluid on structures of the sella turcica and the vestibulocochlear system can modify the pressure of the liquids of the inner ear through the vestibular and cochlear ducts [13].

However, we consider this case a mixed-origin vertigo, considering the most likely etiology for the patient complaint is dependent on a defect of the posterior circulation and an empty sellar syndrome caused by head trauma.

## Conclusions

Although the incidence of SSNHL secondary to vertebrobasilar ischemia is low, it is important to suspect ischemia in this territory and, particularly in the AICA, in patients with bulbar symptoms (such as transitory loss of consciousness) associated with sudden hearing loss and spontaneous vertigo. Vertebrobasilar dolichoectasia is a risk factor that may predispose to ischemic events in this territory. However, we consider this case a mixed-origin vertigo as the most likely etiology for the patient's complaint due to a defect of the posterior circulation and an empty sellar syndrome caused by head trauma. Continuing research on the matter is essential for determining the best diagnostic approach for patients who complain of these kinds of symptoms and for determining the therapeutic options with the optimal outcome.
